# Pervasive seasonal divergence in lagged responses of vegetation growth to compound drought-heat stress on the Tibetan Plateau

**DOI:** 10.3389/fpls.2026.1879954

**Published:** 2026-07-13

**Authors:** Wenjuan Li, Xiuchen Wu, Zekai Meng, Xiang Li, Wenqi Song, Peng Zhao

**Affiliations:** 1 College of Geographic Science, Qinghai Normal University, Xining, China; 2 Department of Health and Environmental Sciences, School of Science, Xi’an Jiaotong-Liverpool University, Suzhou, China; 3 Academy of Plateau Science and Sustainability, People's Government of Qinghai Province & Beijing Normal University, Xining, China; 4 Faculty of Geographical Science, Beijing Normal University, Beijing, China; 5 School of Geosciences, University of South Florida, Tampa, FL, United States

**Keywords:** compound drought-heat stress, lag response, memory effect, Tibetan plateau, vegetation response

## Abstract

**Introduction:**

The Tibetan Plateau (TP) has experienced increasing frequency and intensity of compound drought-heat stress in recent decades accompanying global warming. However, the spatiotemporal pattern and the underlying factors contributing to vegetation growth responses to such compound stress remain poorly understood, particularly across seasons and vegetation types.

**Methods:**

In this study, monthly accumulated precipitation and maximum temperature data from the TerraClimate dataset were used to develop a Compound Drought-Heat Index (CDHI) in the period of 2000-2022. Vegetation growth responses to seasonal CDHI were then investigated using MODIS Normalized Difference Vegetation Index (MODIS/061/MOD13A1) data.

**Results:**

Our results reveal a distinct seasonal divergence in vegetation growth response to compound drought-heat stress. Specifically, approximately 61.57% of the TP exhibited a one-month lagged response to spring CDHI, whereas in summer, around 65.87% of the study region showed an almost immediate response. Vegetation in hotter and drier subregions of the TP exhibits a stronger positive correlation with compound drought-heat stress, and grasslands were more sensitive to compound drought-heat stress than forests. Spring precipitation, soil moisture, spring downward surface shortwave radiation, and spring CDHI exerted memory effects on both spring vegetation responses and subsequent summer vegetation responses to compound drought-heat stress.

**Discussion:**

These findings uncovered a widespread seasonal divergence in the lagged responses of vegetation growth to compound climate extremes and highlighted the importance of cross-seasonal interactions in understanding ecosystem vulnerability to intensifying extreme climate events.

## 1 Introduction

The global mean surface temperature has increased by approximately 1.1 °C since the Industrial Revolution and is projected to increase by more than 3 °C by the end of this century (Ghanbari et al., 2023; Chen et al., 2023; Yin et al., 2023; Armstrong Mckay et al., 2022; Ribes et al., 2021). Climate warming is expected to exacerbate drought stress on vegetation growth through increased atmospheric evaporative demand and altered precipitation patterns (Wu et al., 2018; Li et al., 2024b). A thorough understanding of vegetation growth responses to drought stress is crucial for predicting the stability and vulnerability of vegetation under future climate warming (Du et al., 2025b; Zhang et al., 2025; Vicente-Serrano et al., 2013). Numerous studies have investigated vegetation growth responses to drought stress using the NDVI and drought indices such as the Standardized Precipitation Evapotranspiration Index (SPEI) at regional to global scales, revealing significant spatiotemporal heterogeneity. For instance, vegetation growth in arid regions tends to experience stronger drought-induced suppression (Zhang et al., 2022; Gu et al., 2025). Notably, droughts are often accompanied by heatwaves, and compound drought-heat stress can have more detrimental impacts on vegetation growth than extreme droughts or heatwaves alone (Zscheischler et al., 2020; Duan et al., 2024).

An increasing number of studies are investigating the responses of vegetation growth to compound drought-heat stress across diverse ecoregions (Gampe et al., 2021; Yin et al., 2023). Vegetation growth responses to compound drought-heat stress are strongly dependent on timing. Generally, favorable climatic conditions in spring could promote vegetation growth, however the concomitant increase in transpiration accelerates soil moisture depletion and this soil water deficit can persist into the summer, potentially exacerbating the occurrence of summer droughts (Bastos et al., 2020; Lian et al., 2020). In high-latitude regions, although spring compound drought-heat stress may lead to reduced precipitation, rising temperatures can also trigger the thawing of frozen soil water, which may partially alleviate water stress and even stimulate vegetation growth (Nitzbon et al., 2020). However, compound drought-heat stress occurring in summer is typically characterized by a combination of significantly reduced precipitation and elevated temperatures. This synergistic effect often leads to a pronounced suppression of vegetation growth, implying a seasonal shift in the sensitivity of vegetation growth to the compound drought-heat stress. Nevertheless, the seasonal shift in vegetation growth responses to compound drought-heat stress remains poorly understood, particularly in regions susceptible to extreme climate events.

Vegetation growth responses to variations in environmental factors are not immediate but instead exhibit distinct time lags, a phenomenon known as lagged responses (Wu et al., 2015a; Davis, 1989). Globally, vegetation growth shows a rapid response to temperature variability, with the strongest correlation at a lag time of 0 to 1 month in more than half of the vegetated lands (Chen et al., 2020). In contrast, the effects of precipitation on vegetation growth are often long-lasting, and can extend to seasonal and multi-year timescales (Wang et al., 2023; Zhan et al., 2022). Additionally, vegetation growth responses to climate perturbations vary among vegetation types. For example, grasslands and shrublands generally exhibit faster responses to warming and drying conditions compared to forests (Cleland et al., 2013; Chen et al., 2025; Anderegg et al., 2015), which is mainly attributed to differences in ecosystem structural complexity, species diversity, and rooting depth (Fischer et al., 2019; Li et al., 2019b; Anderegg et al., 2015). Moreover, vegetation growth often shows even more complex lagged behavior under compound drought-heat stress, as concurrent drought and heat can synergistically intensify water and carbon limitations by constraining photosynthesis, transpiration, and hydraulic efficiency (Li et al., 2022; D’Orangeville et al., 2018; Lian et al., 2021; Li et al., 2023; Liu et al., 2025).

Comprehensive understanding of vegetation growth responses to compound drought-heat stress on the Tibetan Plateau is essential for assessing the vulnerability of high-altitude ecosystems under climate warming (Ding et al., 2018; Wu et al., 2025a). The Tibetan Plateau, often referred to as the “Third Pole” (Yang et al., 2014; Yao et al., 2019), has traditionally been recognized as an energy-limited region where vegetation growth is less constrained by water availability (Sun et al., 2019; Chen et al., 2022; Zhang et al., 2024). However, recent rapid warming has substantially intensified soil moisture deficits and atmospheric water demands, which has increased drought risks for vegetation growth. An increasing number of studies have demonstrated that climate warming is driving the expansion of compound drought-heat stress into higher latitudes, higher elevations, and regions experiencing intensified greening (Li et al., 2024b; Yan et al., 2025). Previous studies have identified lagged responses of vegetation growth to temperature and precipitation variations on the TP (Meng and Zhang, 2021; Chen et al., 2023). Nevertheless, a systematic understanding of seasonal divergence in vegetation growth responses to compound drought-heat events is lacking.

This study aims to explore the seasonal and vegetation-type-specific response patterns of vegetation growth to compound drought-heat stress on the Tibetan Plateau. Specifically, we seek to address the following questions: (1) How do vegetation responses to compound drought-heat stress differ across seasons and among major vegetation types on the Tibetan Plateau? (2) What mechanisms drive seasonal shifts in vegetation sensitivity and lagged responses to compound drought-heat stress? (3) Do spring climatic conditions exert a cross-seasonal influence on summer vegetation responses to compound drought-heat stress? This study seeks to provide new insights into the seasonal lag structures of vegetation responses to compound drought-heat stress and to contribute to an improved understanding of climate-vegetation interactions on the Tibetan Plateau.

## 2 Data and methods

### 2.1 Study area

As the “roof of the world”, the Tibetan Plateau (25°-40°N, 74°-104°E) has an average altitude over 4000 m and covers an area of about 2.5 million square kilometers. Its complex topography interacts with multiple climate systems, including the East Asian monsoon, the Indian monsoon, the westerly jet stream and the Siberian high-pressure climate system, which together shape a climate gradient of “warm and humid in the southeast and dry and cold in the northwest” (Wu et al., 2015b). The mean annual precipitation decreases from 1000 mm in the southeast to 200 mm in the northwest, and the mean annual temperature decreases from 10°C to-5°C. The vegetation distribution exhibits a coupled pattern of vertical and horizontal zonation, with forests, grasslands, and deserts successively distributed from southeast to northwest.

### 2.2 Climate and vegetation data

Monthly accumulated precipitation and maximum temperature data were obtained from the TerraClimate dataset for the period of 2000-2022 (Abatzoglou et al., 2018). The TerraClimate dataset has a spatial resolution of 4 km. It has been widely used to assess drought and heat events (Hammond et al., 2022). In this study, seasonal climate variables were calculated by aggregating monthly records into spring (March-May) and summer (June-August) totals for precipitation and means for temperature (Lian et al., 2020).

Vegetation growth was characterized using the NDVI. The MODIS-NDVI product (MODIS/061/MOD13A1) used in this study was acquired from Google Earth Engine (Beck et al., 2006; Didan, 2015). This dataset provides NDVI during the period of 2000–2022 with a 500 m spatial resolution and 16 days temporal resolution. The Maximum Value Composite method was applied to generate monthly NDVI values, which were then averaged to obtain seasonal means for spring (March-May) and summer (June-August). To ensure data quality, Quality Assurance flags provided with the MOD13A1 product were used to exclude pixels affected by cloud contamination, snow cover, or water bodies. The NDVI time series is characterized by periodic variation and can be decomposed into three components: a long-term trend component, a seasonal component, and a residual component (Watts and Laffan, 2014, Jiang et al., 2024). In this study, we applied Seasonal-Trend decomposition using LOESS to extract the residual component of the NDVI time series, as described in Equation 1:

(1) $$\begin{array}{c} Y=Trend+Season+Residual \end{array}$$

Where Trend represents the long-term trend, Season represents seasonal variations, and Residual represents the remaining component. The residual component was then used in the subsequent analysis to characterize vegetation responses to compound drought-heat stress.

To thoroughly investigate the factors contributing to modelled vegetation responses to compound drought-heat stress in spring and summer, we incorporated a wide range of environmental factors including climate, soil texture, and topography. In terms of climate factors, we considered the seasonal mean Actual Evapotranspiration (AET), Soil Moisture (SM), Maximum Temperature (TMMX), Downward surface shortwave radiation (SRAD) and Vapor Pressure Deficit (VPD). Gridded monthly AET, SM, TMMX, SRAD and VPD were obtained from the TerraClimate dataset at a spatial resolution of 4 km (Zhao et al., 2023). Gross Primary Productivity (GPP) data were derived from the NASA Terra MODIS sensor as an 8-day composite data. Vegetation type data were obtained from the MODIS/061/MCD12Q1 dataset (Friedl and Sulla-Menashe, 2022) with a spatial resolution of 500 m. The Digital Elevation Model (DEM) data were derived from NASADEM with a spatial resolution of 30 m (Xu et al., 2024a). Sand content data (SoilGrids, Sand) were sourced from the WoSIS database at a spatial resolution of 250 m. The Vegetation type data, GPP, DEM and soil sand content were resampled into a spatial resolution of 4 km using nearest-neighbor assignment method. The GPP data were composited using the Maximum Value Composite method to generate monthly GPP values. Except for the static environmental data, including DEM, sand content data, and vegetation type data, all other climate variables were averaged over spring (March-May) and summer (June-August) to reflect seasonal variations, seasonal precipitation was calculated as cumulative totals. All the above datasets were obtained via Google Earth Engine.

### 2.3 Calculation of the compound drought-heat index

In this study, a Compound Drought-Heat Index (CDHI) was constructed to quantify the joint severity of drought and heat events. CDHI provides a standardized and comparable measure of drought-heat severity and has advantages over traditional single-variable indices such as SPI or SPEI (Hao et al., 2020, Hao et al., 2019). The monthly CDHI was calculated using the monthly Standardized Precipitation Index (SPI) and the monthly Standardized Temperature Index (STI) (Hao et al., 2021; Xu et al., 2024b; Zhou et al., 2024; Wu et al., 2020). The monthly SPI was calculated by fitting the Gamma distribution to obtain the probability density function F(x) of monthly precipitation. This marginal distribution was then converted to a standard normal distribution $\Phi^{-1}(F(x))$ (Equation 2) (McKee et al., 1993).

(2) $$\begin{array}{c} SPI=\Phi^{-1}(F(x)) \end{array}$$

Monthly STI was calculated in a similar way to monthly SPI, but using monthly mean temperature data instead of precipitation data (Zscheischler et al., 2014; Duan et al., 2024).

The Copula model was introduced to decouple the variables’ marginal distributions from their dependence structure, thereby enabling precise characterization of complex features including correlations, asymmetries, and other nonlinear dependencies within the joint probability distribution (i.e., SPI and STI) (Wu et al., 2021). Several Copula families exist (e.g., Archimedean, Elliptical, Extreme Value), each with different dependence characteristics (Wang et al., 2020; Wu and Hu, 2023). In this study, we compared five bivariate Copula families, namely Gaussian, Student’s t, Gumbel, Clayton, and Frank Copulas, for modeling the dependence between SPI and STI. The parameters of each Copula were estimated using maximum likelihood estimation. The goodness of fit was evaluated using AIC, BIC, and *RMSE* (Cheng et al., 2023; Hao et al., 2019; Zscheischler and Seneviratne, 2017) (**Table 1**). According to the evaluation, this study applies the Gaussian Copula model to calculate the joint distributions of SPI and STI (Xiang et al., 2024; Gao et al., 2025), as shown in Equation 3:

**Table 1:** Goodness-of-fit evaluation for different copula families.

Copula	AIC	BIC	RMSE
Gaussian	-39785.17	-39774.05	0.0133
Student t	-246.21	-234.2	unstable
Gumbel	2	13.12	0.0278
Clayton	-14680.12	-14669	0.0219
Frank	unstable	unstable	0.0954

(3) $$\begin{array}{c} p_{CDHI}=P(X_{SPI}<x_{SPI},Y_{\left(-STI\right)}<y_{\left(-STI\right)})=C_{n}(u,v) \end{array}$$

Where u  and  v  are marginal distributions of random variables X and Y, and $C_{n}(u,v)$ is the Copula function suitable for SPI and STI. The joint distribution $p_{CDHI}$ is derived to quantify the severity of concurrently occurring drought and heat events.

The joint distribution $p_{CDHI}$ was transformed into a standardized index by fitting $\;F(p_{CDHI})$ and then converting it to a standard normal variable CDHI (Equation 4) (Xiang et al., 2024):

(4) $$\begin{array}{c} CDHI=\Phi^{-1}(F(p_{CDHI})) \end{array}$$

A lower CDHI value indicates greater severity of the compound drought-heat stress. The calculation of CDHI was conducted in Python 3.9.

### 2.4 Identification of the lag time in the vegetation responses to CDHI

To quantify the temporal lag in vegetation growth responses to compound drought-heat stress, pixel-wise Pearson correlation coefficients were calculated between the CDHI time series and NDVI residual component under different temporally shifted three-month response windows. For each pixel, the CDHI series was correlated with NDVI derived from different temporally shifted three-month windows, following previous studies showing that vegetation growth responses to drought rarely exceed a four-month delay across large spatial scales (Wang et al., 2023; Zhan et al., 2022; Zuo et al., 2021). Given that vegetation growth responds to climate variations more rapidly in summer, the window shift was restricted to 0–2 months for summer analyses (Yuan et al., 2024; Zuo et al., 2021; Li et al., 2024a). The identification of time lag in vegetation growth responses to CDHI was estimated using the following Equations 5 and 6.

(5) $$\begin{array}{c} R_{t}=Corr(CDHI,NDVI_{t}) \end{array}$$

(6) $$\begin{array}{c} R_{lag}=\max(Rt) \end{array}$$

where,  CDHI represents the seasonal mean Compound Drought-Heat Index, ${\text{NDVI}}_{\text{t}}\;$represents the mean NDVI residual component within the three-month response window shifted by  t  month. In spring, the March-May mean CDHI was correlated with the mean NDVI residual component for March-May, April-June, May-July, and June-August, corresponding to window shifts of 0, 1, 2, and 3 months, respectively. In summer, the June-August mean CDHI was correlated with the mean NDVI residual component for June-August, July-September, and August-October, corresponding to window shifts of 0, 1, and 2 months, respectively. $\;R_{t\;}$ is the Pearson correlation coefficient, where $\;R_{lag\;}$ represents the strongest correlation coefficient and the corresponding t  is defined as the lag time. We further characterize the spatial patterns of lag time along climatic gradients, defined by the Mean Annual Temperature (MAT) and Mean Annual Precipitation (MAP).

### 2.5 Factor importance for vegetation response to CDHI based on XGBoost

To identify the key environmental factors influencing vegetation growth responses to CDHI, an eXtreme Gradient Boosting (XGBoost) regression model was employed, coupled with Shapley Additive exPlanations (SHAP) for model interpretation (Meng et al., 2024; Futagami et al., 2021). The XGBoost model was trained in Python using XGBRegressor from the *xgboost* package, and SHAP analysis was performed using the *shap* package. The target variable was the maximum correlation coefficient $\;R_{lag}\;$ between NDVI residual component and CDHI. During data preprocessing, all samples containing missing values were removed to ensure model validity and stability. Categorical variables were converted into integers using label encoding to meet XGBoost’s requirements for numerical inputs. The feature variables and target variables were partitioned, with the dataset randomly split into a training set (75%) and a test set (25%) to evaluate model performance. A stepwise grid search combined with five-fold cross-validation was implemented to iteratively optimize key hyperparameters of the XGBoost model. Specifically, maximum tree depth (max_depth), number of boosting trees (n_estimators), and learning rate (learning_rate) were tuned sequentially, while other parameters were kept fixed. During hyperparameter tuning, five-fold cross-validation was performed on the training set using negative root mean squared error as the scoring criterion, and the parameter combination with the lowest cross-validated *RMSE* was selected. Finally, the optimal parameters were incorporated into the XGBoost model (max_depth=9, n_estimators=300, and learning_rate=0.1), and model performance was evaluated on both the training and testing datasets using *RMSE* and *R²*. To enhance model transparency and interpretability, the SHAP method (Lundberg and Lee, 2017) was applied to interpret the model outputs. SHAP values were computed for each feature and each sample. A scatter plot was then generated to display the top nine most influential features ranked by their relative contributions. Furthermore, Locally Weighted Scatterplot Smoothing (LOWESS) curves were fitted to continuous variables to illustrate nonlinear relationships (Wu et al., 2025b).

## 3 Results

### 3.1 Spatiotemporal patterns in vegetation growth responses to CDHI

**Figure 1:**
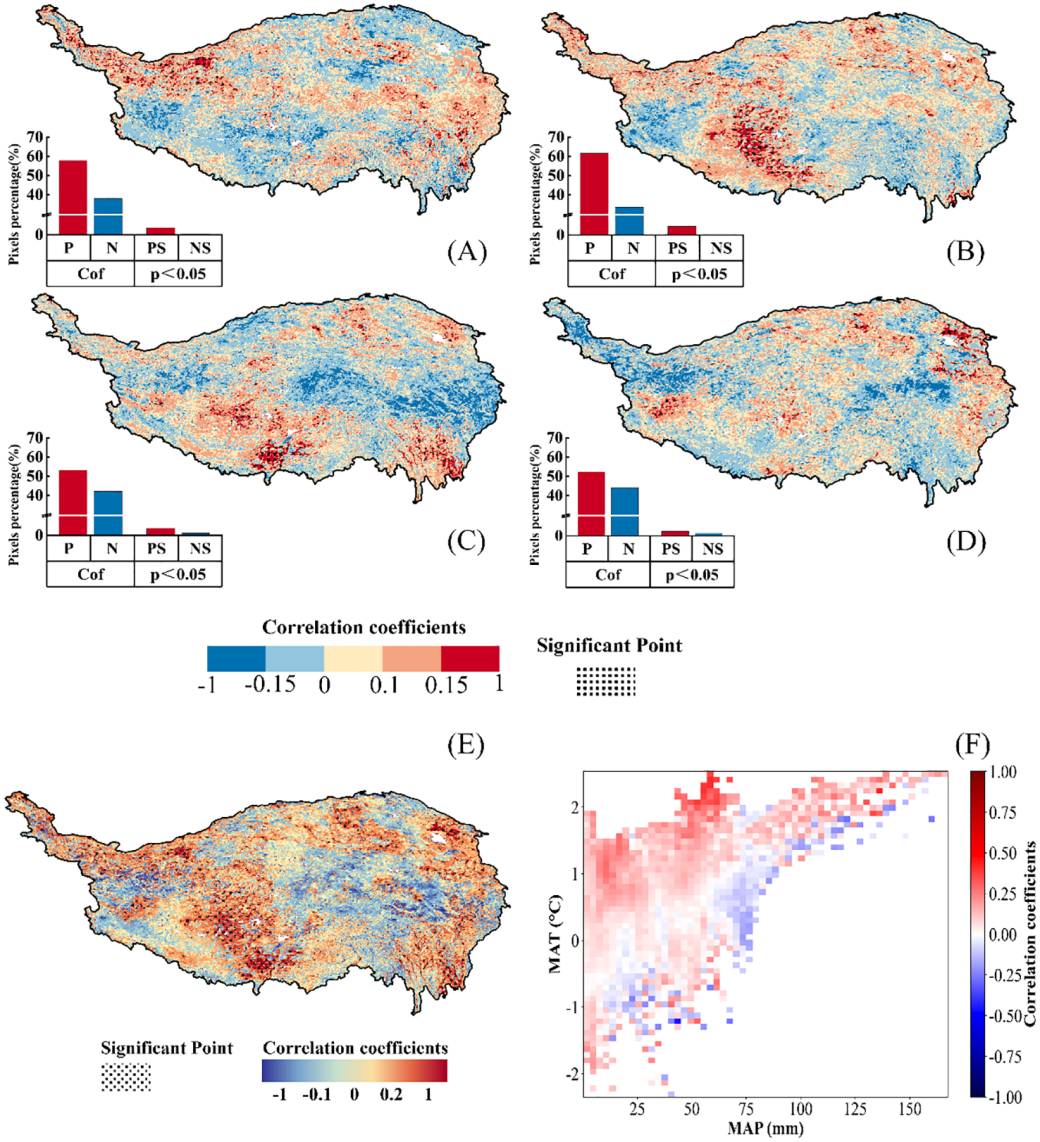
Multi-dimensional lag effects of spring vegetation responses to compound drought-heat stress. **(A–D)** Spatial patterns of the correlation between spring CDHI and NDVI at lag intervals of 0 to 3 months. **(E)** Spatial distributions of the maximum correlation coefficient $R_{lag}$ between the CDHI and lagged NDVI in spring. **(F)** Climatic space diagrams illustrating the joint effects of mean annual precipitation (MAP) and mean annual temperature (MAT) on $R_{lag}$ for spring. In the figure, P represents the proportion of pixels with positive correlation to the total number of pixels, N represents the proportion of pixels with negative correlation to the total number of pixels, PS represents the proportion of pixels with significant positive correlation to the total number of pixels, and NS represents the proportion of pixels with significant negative correlation to the total number of pixels.

**Figure 2:**
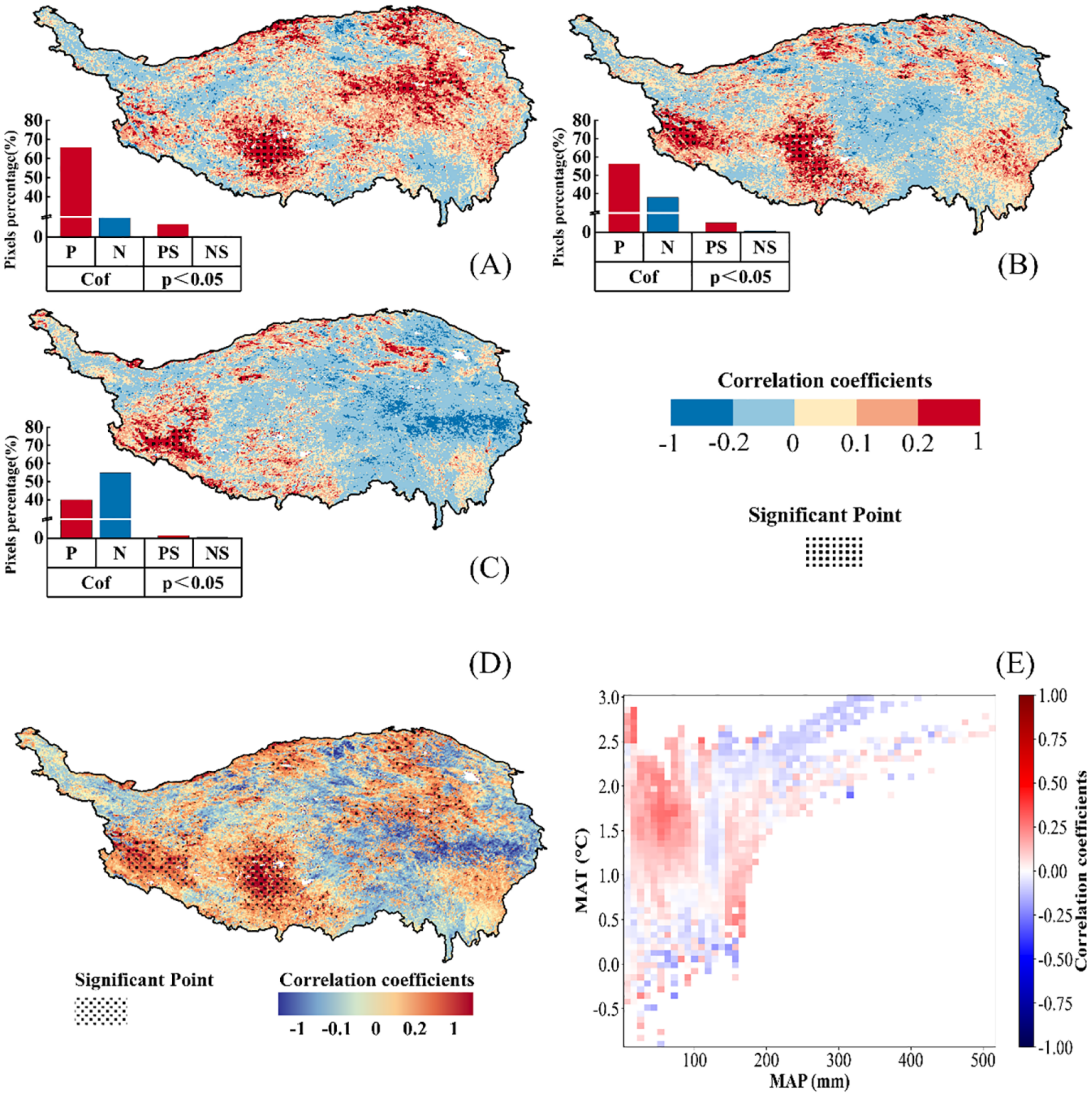
Multi-dimensional lag effects of summer vegetation responses to compound drought-heat stress. **(A–C)** Spatial patterns of the correlation between summer CDHI and NDVI at lag intervals of 0 to 2 months. **(D)** Spatial distributions of the maximum correlation coefficient $R_{lag}$ between the CDHI and lagged NDVI in summer. **(E)** Climatic space diagrams illustrating the joint effects of mean annual precipitation (MAP) and mean annual temperature (MAT) on $R_{lag}$ for summer. In the figure, P represents the proportion of pixels with positive correlation to the total number of pixels, N represents the proportion of pixels with negative correlation to the total number of pixels, PS represents the proportion of pixels with significant positive correlation to the total number of pixels, and NS represents the proportion of pixels with significant negative correlation to the total number of pixels.

Across the Tibetan Plateau, the response of vegetation growth to CDHI exhibits pronounced seasonal divergence in both spatial pattern and mean time lags. In spring, 61.57% of the study region exhibited a positive correlation between vegetation growth and CDHI at one-month lag, with 2.67% showing significantly (*p* < 0.05) positive correlation. Pronounced positive responses were located in the southwestern region. The proportions of regions with positive correlation were lower at time lags of 0, 2, and 3 months (57.8%, 53.09%, and 52.3%, respectively). The regions exhibiting significantly positive correlations at different time lags were also concentrated in the southwestern, southeastern, and northeastern parts of the Tibetan Plateau (**Figures 1A–E**). In summer, 65.87% of the Tibetan Plateau shows a positive correlation with CDHI at time lag 0, with 4.17% showing significantly positive correlations, and this fraction decreases at time lags of 1 and 2 months to 56.23% and 40.07%, respectively. Significantly positive correlations are predominantly concentrated in the southwestern and northeastern Tibetan Plateau across all time lags (**Figures 2A–D**). Pixels showing negative correlations were mainly concentrated in the central and western Tibetan Plateau, but these negative relationships were generally not statistically significant. Spatial pattern analysis within a climate space further revealed that vegetation growth tended to exhibit stronger positive correlations with CDHI in regions characterized by lower MAP and higher MAT in both spring and summer (**Figures 1F**, **2E**).

### 3.2 Seasonal variation in vegetation growth responses to CDHI between vegetation types

**Figure 3:**
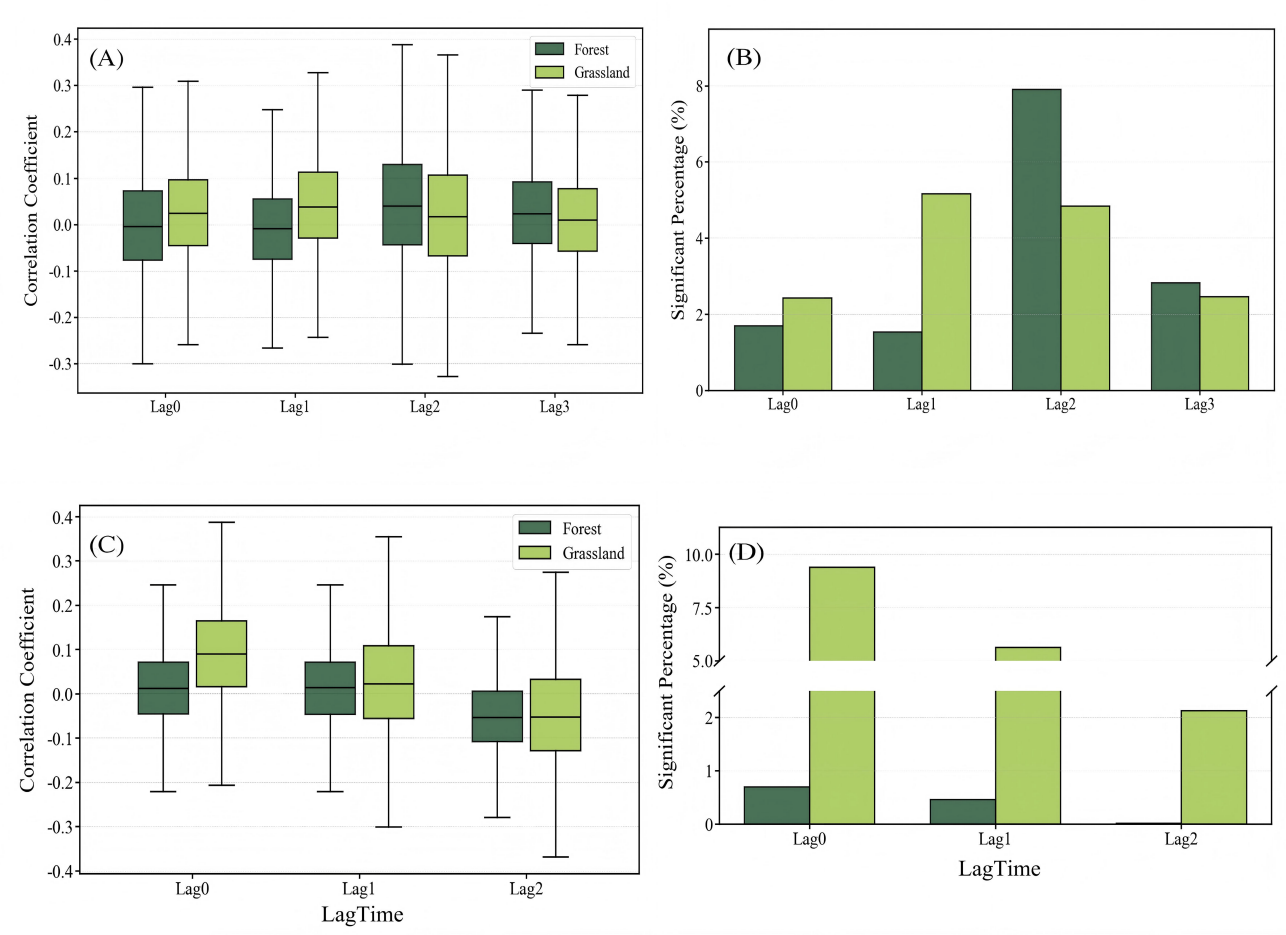
Seasonal response differences of different vegetation types to compound drought-heat stress. **(A)** Mean correlation coefficients of pixels showing significant positive correlation for each vegetation type during spring. **(B)** Changes in the proportion of significantly positively correlated pixels relative to the respective total number of pixels for forests and grasslands across different lag times in spring. **(C)** Mean correlation coefficients of pixels showing significant positive correlation for each vegetation type during summer. **(D)** Changes in the proportion of significantly positively correlated pixels relative to the respective total number of pixels for forests and grasslands across different lag times in summer.

The lagged responses of vegetation growth to CDHI differed between forests and grasslands (**Figure 3**). The analysis included 6,030 forest pixels and 104,358 grassland pixels. Both forests and grasslands exhibit a clear lagged response to spring CDHI, but differed in mean time lags (**Figures 3A, B**). For forests, the dominant response occurs at a time lag of approximately two months. In contrast, a slightly higher proportion with maximum correlation coefficient was observed at one-month time lag in grasslands. Interestingly, both forests and grasslands exhibited immediate responses to summer CDHI, with the highest proportions of significantly positive correlation occurring at time lag 0 (**Figures 3C, D**). Grassland vegetation showed stronger responses to CDHI than forest vegetation in summer, as indicated by much higher correlation coefficients across all time lags.

### 3.3 Factors contributing to modelled vegetation responses to CDHI

**Figure 4:**
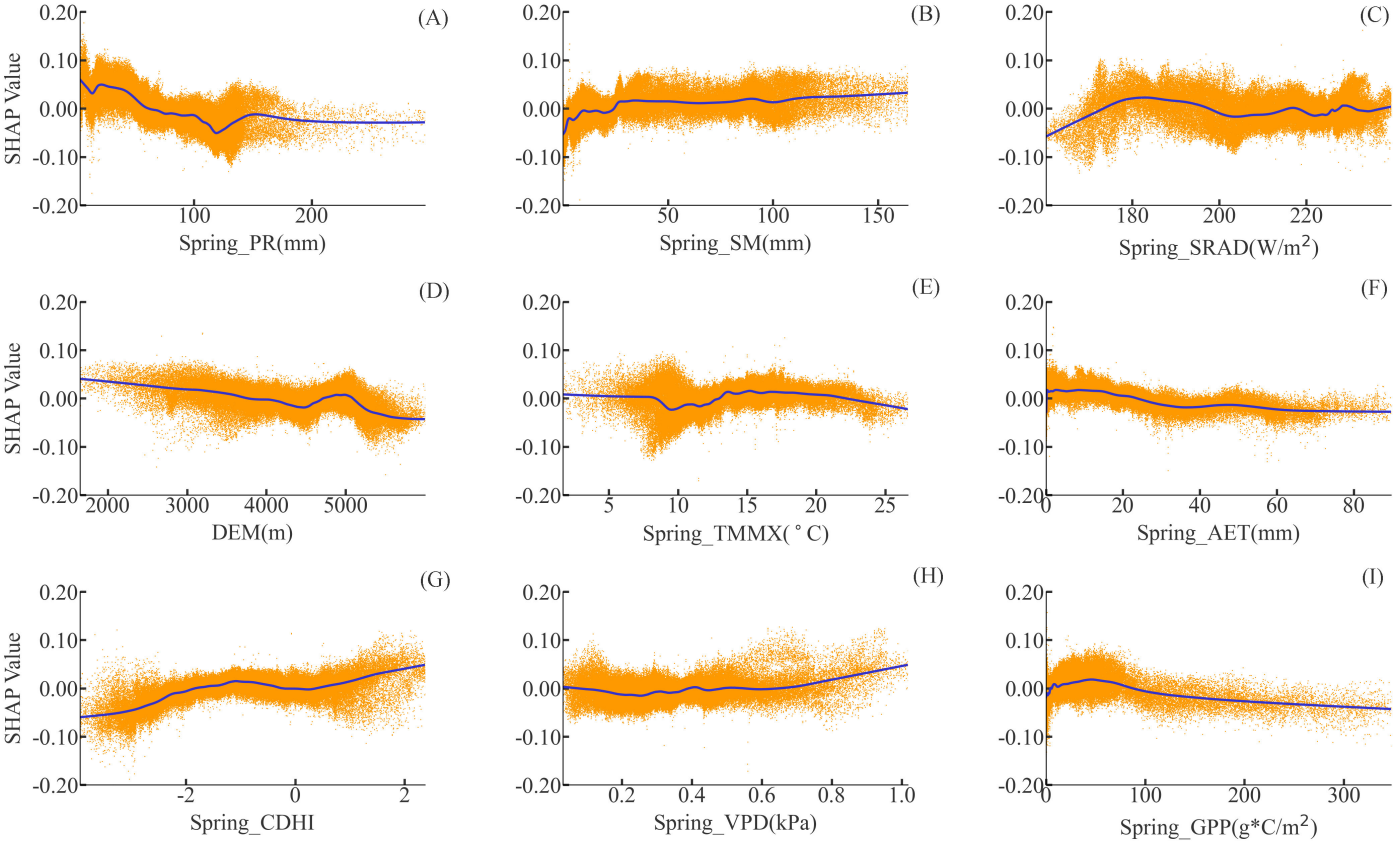
Importance ranking of selected variables affecting vegetation sensitivity to compound drought-heat stress in spring. **(A)** Accumulated precipitation. **(B)** Soil moisture. **(C)** Downward surface shortwave radiation. **(D)** Digital Elevation Model. **(E)** Maximum temperature. **(F)** Actual evapotranspiration. **(G)** Compound drought-heat Index. **(H)** Vapor pressure deficit. **(I)** Gross primary productivity.

The XGBoost models performed well in both spring and summer, with *R²* values of 0.44 and 0.54, *RMSE* values of 0.13 and 0.12, respectively. Across both spring and summer, the precipitation (Spring_PR and Summer_PR) was consistently ranked among the most important individual predictors (**Figure 4A**, **5A**). The SHAP values for spring and summer precipitation generally decrease as precipitation increases; under low precipitation conditions, they show a positive contribution to ( $R_{lag}$), whereas under higher precipitation conditions, they switch to a negative contribution.

**Figure 5:**
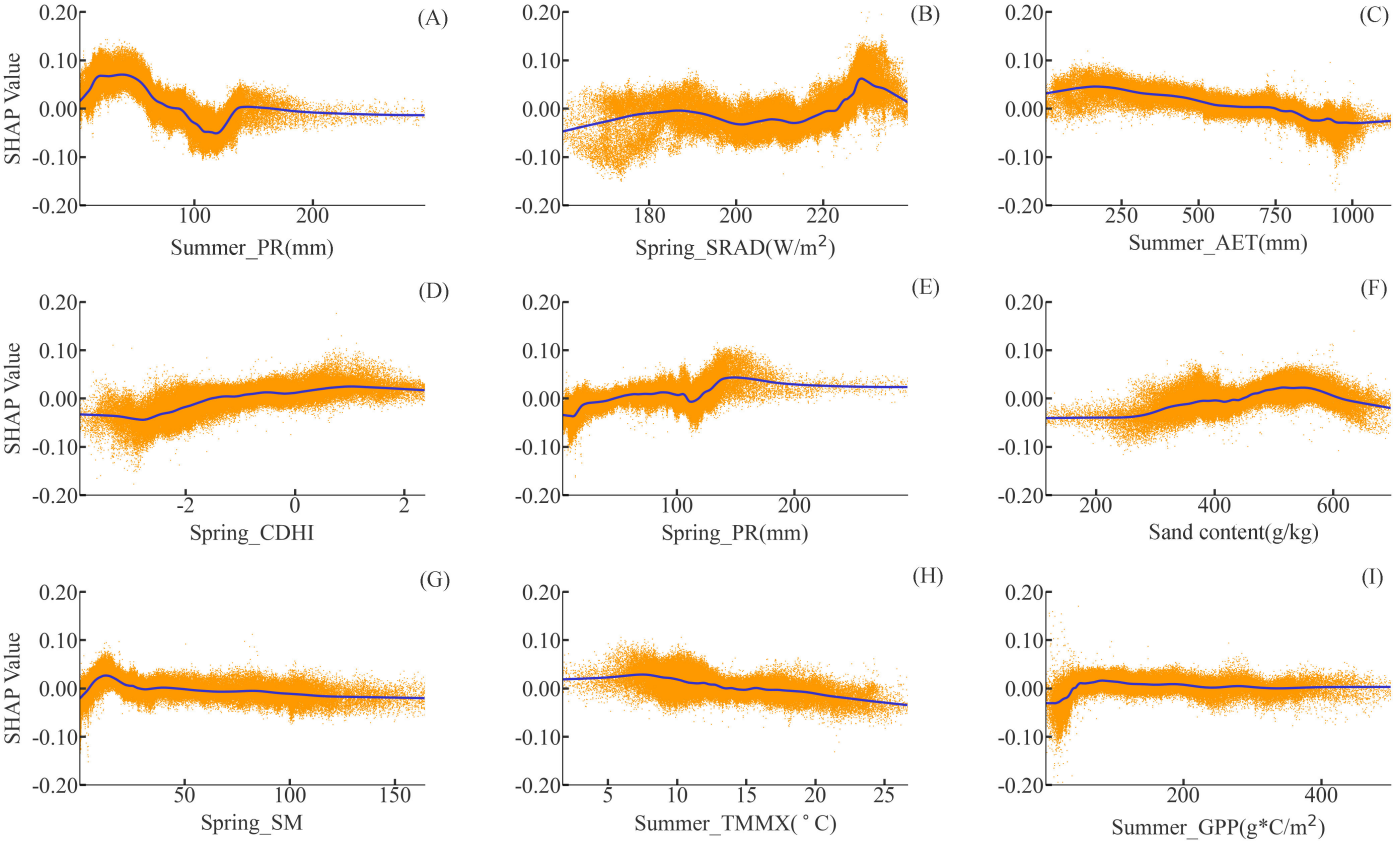
Importance ranking of selected variables affecting vegetation sensitivity to compound drought-heat stress in summer. **(A)** Summer accumulated precipitation. **(B)** Spring downward surface shortwave radiation. **(C)** Summer actual evapotranspiration. **(D)** Spring compound drought-heat Index. **(E)** Spring accumulated precipitation. **(F)** Sand content (0–5 m). **(G)** Spring soil moisture. **(H)** Summer maximum temperature. **(I)** Summer gross primary productivity.

The other factors controlling vegetation growth responses to CDHI also exhibited seasonal differences. In spring, the SHAP values for Spring_SM, Spring_CDHI, and Spring_VPD slightly increased with increasing values of these variables (**Figures 4B, G, H**). In contrast, the SHAP values for DEM, Spring_AET and Spring_GPP generally decreased as their values increased (**Figures 4D, F, I**). Spring_SRAD and Spring_TMMX exhibit a distinct nonlinear relationship, both showing a strong positive contribution to $\left(R_{lag}\right)$ within the intermediate ranges, while their contributions are weaker under lower or higher conditions (**Figures 4C, E**). In summer, the SHAP values for Spring_SRAD, Spring_CDHI, and Spring_PR increased with increasing values of these variables (**Figures 5B, D, E**). By contrast, the SHAP values for Summer_AET, Spring_SM, and Summer_TMMX generally decreased as the variables increased (**Figures 5C, G, H**). The influence of sand content exhibits a nonlinear pattern, with higher SHAP values at intermediate sand content and weaker contributions under lower or higher sand content (**Figure 5F**). Summer_GPP has the lowest overall contribution and an unstable response relationship (**Figure 5I**).

## 4 Discussion

### 4.1 Seasonal divergence in vegetation growth responses to CDHI

This study reveals clear seasonal divergence in the responses of vegetation growth to compound drought-heat stress. The analyses indicate that vegetation growth responds to CDHI more rapidly in summer than in spring (**Figure 1B**, **2A**). This seasonal divergence stems from the core seasonal features of vegetation-climate interactions. In spring, low temperatures are often associated with low soil temperature and slow frozen soil thawing, which suppress microbial activity and slow nutrient release (Yang et al., 2025). Although compound drought-heat stress may accelerate snowmelt and soil thawing, thereby releasing additional water that can partially offset the direct impacts of drought (Huang et al., 2019; Li et al., 2020), this transient water supply does not immediately translate into vegetation growth. Instead, the delayed activation of soil microbial communities and the slow recovery of root activity following soil thawing create lagged biogeochemical feedback prolonging the time required for vegetation to respond to the compound stress. On the other hand, the melting of frozen soil in spring is a process of heat accumulation, and a lag response clearly occurs when vegetation absorbs water from meltwater to resist the water deficit caused by compound drought-heat stress (**Figure 4B**). This highlights that the lagged response is jointly controlled by the initial thermal limitation and the post-thaw recovery pace of belowground processes.

In summer, thermal conditions become largely sufficient or even excessive, the rise in atmospheric vapor pressure deficit (VPD) renders the ecosystem more sensitive to fluctuations in soil moisture (Chen et al., 2024; Du et al., 2025a). When compound drought-heat stress occurs during this period, dramatic escalation in temperatures and reduced precipitation intensify atmospheric aridity, leading to rapid soil moisture decline (Xu et al., 2021; Zhou et al., 2019). High VPD normally drives stronger vegetation transpiration; however, concurrent soil water limitations force stomatal closure, which in turn reduces transpiration and suppresses local precipitation through land atmosphere feedbacks, further exacerbating the spatial extent and severity of the compound stress (Seneviratne et al., 2010; Teuling et al., 2010; Chen et al., 2024). Unlike in spring, where thermal constraints and post-thaw biogeochemical lags buffer vegetation response, summer vegetation operates under near-instantaneous water availability control. Once soil moisture drops, vegetation functioning is directly and immediately impaired, leaving little room for buffering or lagged responses. Thus, the short response lag in summer is mechanistically attributed to the combination of dominant water limitation, rapid atmospheric drying via elevated VPD, and tight land-atmosphere coupling that accelerates and amplifies the compound stress.

Pixels showing negative correlations were mainly distributed in the central and northwestern Tibetan Plateau. These regions are generally characterized by high elevation, cold climatic conditions, and sparse vegetation, where vegetation growth may be more limited by low temperature than by water availability alone (Zhu et al., 2023; Li et al., 2019a). Under such cold high-elevation conditions, higher temperatures may favor vegetation growth by improving thermal conditions, extending the growing season, enhancing photosynthetic activity, promoting snowmelt, and accelerating soil thawing (Huang et al., 2019; Li et al., 2020).

### 4.2 Greater vulnerability of vegetation growth to CDHI in warmer and drier regions

In the Tibetan Plateau region, the impact of compound drought-heat stress on vegetation growth exhibits significant spatial heterogeneity, with vegetation in the southwest and northeast regions showing higher sensitivity to CDHI (**Figures 1**, **2**). This is consistent with previous findings (Zhang et al., 2023; Wu et al., 2024; Ding et al., 2018). Previous studies have reported a declining precipitation trend in both regions of the Tibetan Plateau over recent decades, leading to persistently severe drought conditions that have intensified temporally (Wang et al., 2021). The vegetation in the southwest and northeast regions is primarily composed of herbaceous plants, and the ecosystem structure is relatively simple (Li et al., 2019b). High VPD directly leads to faster stomatal closure in grasslands, causing photosynthesis to enter an inhibited state earlier (Yuan et al., 2019; Grossiord et al., 2020). Moreover, herbaceous plants have small biomass and relatively limited non-structural carbohydrates reserves. Under drought and heat stress, the high respiratory consumption brought by high temperatures will quickly deplete their internal carbon reserves, causing them to face danger earlier (Deng et al., 2021). At the same time, shallow-rooted herbaceous vegetation mainly relies on surface water to adapt to drought conditions. High temperatures accelerate the evaporation of surface soil moisture, due to the lack of deep-water replenishment, the water supply for grasslands will rapidly deplete, making them more sensitive to compound drought-heat stress (Gampe et al., 2021; Liu et al., 2024). In addition, the southwest region of the Tibetan Plateau is mountainous with a high average altitude and little cloud cover. The coupling of intense solar radiation and high temperatures makes the transpiration pressure on the vegetation surface far exceed its water supply capacity. Furthermore, the southwest region is characterized by the distribution of permafrost or seasonal frozen soil. Compound drought-heat stress accelerates the thawing of the active layer (Zhao et al., 2021). Although snowmelt may provide replenishment in the short term, in the long run, surface water will subsequently infiltrate into deeper layers. However, the soil texture is mostly alpine desert soil with low organic matter content and poor available water capacity, resulting in limited and depleted surface water accessible to shallow-rooted vegetation (Jiang et al., 2020). On the other hand, the northeastern Tibetan Plateau is located in a climatic transition zone, meaning that precipitation here is greatly influenced by fluctuations in monsoon intensity. A slight retreat or weakening of the monsoon leads to a sudden drop in precipitation (Huang et al., 2023). There exists a precipitation threshold for vegetation growth. The background precipitation in the northeast region is precisely near this critical point, and any slight climate change leading to precipitation anomalies will enhance the sensitivity of vegetation to compound drought-heat stress (Shen et al., 2015).

### 4.3 Seasonal divergence in the importance of factors associated with vegetation responses to CDHI

Our findings indicate that the important factors behind vegetation responses to compound drought-heat stress exhibit distinct seasonal divergence between spring and summer. In spring, Spring_PR, Spring_SM and Spring_SRAD were identified as the most important predictors (**Figures 4A–C**), suggesting that the response of vegetation growth to compound drought-heat stress during the early growing season was jointly regulated by water supply, soil moisture availability, and energy conditions. Moreover, the increasing SHAP values under higher VPD conditions in spring indicate that vegetation responses to CDHI became stronger under relatively dry atmospheric conditions (**Figure 4H**). Together with the negative contribution of higher precipitation and the important role of solar radiation, these results suggest that vegetation growth was more strongly coupled with CDHI under low-precipitation, high-radiation, and low-humidity conditions. This finding indicates that vegetation growth is more vulnerable to CDHI in warmer and drier regions (Wankmüller et al., 2024). Second, the negative impacts of DEM suggest that high-altitude regions of the Tibetan Plateau are typically characterized by low temperatures, high snow cover, and sparse vegetation; vegetation growth may be more constrained by thermal conditions and soil microbial metabolism, thereby strengthening the negative correlation between CDHI and NDVI (**Figure 4D**) (Davidson and Janssens, 2006). In summer, the decreased relative importance of DEM and seasonal mean maximum temperature, together with the increased importance of Summer_AET and Sand content, suggests a clearly seasonal shift in the factors associated with vegetation responses. As the growing season progresses into summer, thermal limitation may become less dominant, whereas water consumption processes and soil water-holding capacity may play a more important role in shaping the responses of NDVI to CDHI. Lower AET is generally observed in regions characterized by unfavorable hydrothermal conditions and unstable vegetation growth. Consequently, when subjected to compound drought-heat stress, vegetation in these areas tends to exhibit a stronger response than that in higher-AET regions. (**Figure 5C**) (Lieth, 1975). Sand content exhibited the highest SHAP values at intermediate levels, where vegetation tends to experience stronger CDHI-induced stress due to its modulation of aeration and drainage. Conversely, its contribution weakened at extreme sand fractions, as vegetation responses become more dependent on soil water-holding capacity and local hydrothermal conditions (**Figure 5F**) (González-Zamora, 2021; Jiang et al., 2020).

Another important finding is the memory effects of Spring_PR, Spring_SM, Spring_SRAD and Spring_CDHI on the vegetation growth responses to compound stress in summer, which is probably associated with deep soil water storage and plant ecophysiological processes (Ogle et al., 2015; Richard et al., 2008; Lian et al., 2021) (**Figures 5B, D, E, G**). Spring precipitation and soil moisture constitute the physical “reservoir” for the survival of summer vegetation. In the alpine ecosystems of the Tibetan Plateau, effective water formed by spring precipitation and snowmelt in certain areas can penetrate into the deep soil. When summer compound stress hits and the surface soil dries up rapidly, the deep water reserved in spring can, to a certain extent, alleviate the impact of compound drought-heat stress on vegetation through the hydraulic lift action of roots. In addition, spring downward surface shortwave radiation contributes to the accumulation of non-structural carbohydrates in vegetation; these energy reserves can maintain the osmotic adjustment ability and tissue survival of vegetation cells when summer compound stress leads to photosynthetic inhibition, thereby affecting the response of summer vegetation to compound drought-heat stress (Thalmann and Santelia, 2017). The memory effect of spring CDHI suggests that the combined hydrothermal conditions in spring may be associated with summer vegetation responses to compound drought-heat stress. This seasonal shift indicates that vegetation responses to compound drought-heat stress on the Tibetan Plateau may exhibit clear seasonal differences and cross-seasonal memory effects.

### 4.4 Strengths and limitations

Although this study provides insights into seasonal and vegetation-type differences in vegetation growth responses to compound drought-heat stress, several limitations should be acknowledged. First, because NDVI systematically saturates at high Leaf Area Index (LAI) levels, it reduces the apparent sensitivity of forest ecosystems to compound drought-heat stress (CDHI), which may introduce uncertainties into the comparative analysis across different vegetation types. Second, the XGBoost model was used to identify environmental factors associated with variations in the strength of vegetation responses to CDHI. However, we mainly considered external environmental factors, while excluding intrinsic vegetation characteristics, including vegetation structure, non-structural carbohydrates, and rooting depth. This may partly explain the relatively low *R²* of the model. Third, our correlation analysis quantified the relationship between compound drought-heat stress and vegetation responses but did not account for the potential nonlinearities between these variables. Finally, the study focused primarily on the dominant vegetation types on the Tibetan Plateau, whereas less abundant vegetation types were not included, which may limit the comprehensiveness of the findings.

Therefore, future studies should incorporate alternative vegetation proxies that are less prone to saturation, such as Enhanced Vegetation Index (EVI) or Solar-Induced Chlorophyll Fluorescence (SIF), to better disentangle structural effects from true physiological responses. In addition, more comprehensive influencing factors, such as human activities, pest and disease disturbances, vegetation structure, and rooting depth, should be incorporated to improve our understanding of vegetation responses to compound drought-heat stress. Furthermore, employing a variety of machine learning methods could help uncover complex, potential nonlinearities among relationships and enhance the exploration of underlying mechanisms.

## 5 Conclusions

This study comprehensively evaluated the potential divergence in vegetation growth responses to compound drought-heat stress between spring and summer on the Tibetan Plateau. The results revealed pronounced seasonal contrasts in vegetation sensitivity to compound drought-heat stress. In spring, both grasslands and forests exhibited lagged responses, but the dominant lag differed between vegetation types, with grasslands mainly showing an approximately one-month lag and forests showing an approximately two-month lag. In contrast, vegetation responded more rapidly to compound drought-heat stress in summer. In addition, spring precipitation, soil moisture, spring downward surface shortwave radiation and CDHI exhibited significant memory effects, likely driven by deep soil water storage and plant ecophysiological processes, influencing vegetation sensitivity to compound drought-heat stress in the subsequent season. Overall, our findings demonstrate that vegetation responses to compound drought-heat stress exhibit significant seasonal and vegetation type variations across the Tibetan Plateau, which are intrinsically regulated by environmental memory effects. This highlights that future research on ecological vulnerability must incorporate these cross-seasonal hydrothermal dynamics to provide a solid scientific foundation for formulating precise and diversified adaptive management strategies for high-altitude ecosystems.

## Data Availability

The datasets analyzed for this study can be found in the Google Earth Engine Data Catalog. Specifically, the TerraClimate dataset is available at [https://developers.google.com/earth-engine/datasets/catalog/IDAHO_EPSCOR_TERRACLIMATE]; the MODIS Normalized Difference Vegetation Index (MOD13A1) dataset is available at [https://developers.google.com/earth-engine/datasets/catalog/MODIS_061_MOD13A1]; and the MODIS Land Cover Type (MCD12Q1) dataset is available at [https://developers.google.com/earth-engine/datasets/catalog/MODIS_061_MCD12Q1]. Further inquiries can be directed to the corresponding author.
